# A Risk-Averse Shelter Location and Evacuation Routing Assignment Problem in an Uncertain Environment

**DOI:** 10.3390/ijerph16204007

**Published:** 2019-10-19

**Authors:** Bian Liang, Dapeng Yang, Xinghong Qin, Teresa Tinta

**Affiliations:** 1 School of Economics & Management, Tongji University, Shanghai 200092, China; 1730316@tongji.edu.cn; 2 School of Business Planning, Chongqing Technology and Business University, Chongqing 400067, China; qinxh@ctbu.edu.cn; 3 Department of Geographical Sciences, University of Maryland, College Park, MD 20742, USA; ttinta@terpmail.umd.edu

**Keywords:** shelter location, traffic assignment, disaster management, stochastic programming, risk aversion, uncertainty

## Abstract

Disasters such as hurricanes, earthquakes and floods continue to have devastating socioeconomic impacts and endanger millions of lives. Shelters are safe zones that protect victims from possible damage, and evacuation routes are the paths from disaster zones toward shelter areas. To enable the timely evacuation of disaster zones, decisions regarding shelter location and routing assignment (i.e., traffic assignment) should be considered simultaneously. In this work, we propose a risk-averse stochastic programming model with a chance constraint that takes into account the uncertainty in the demand of disaster sites while minimizing the total evacuation time. The total evacuation time reflects the efficacy of emergency management from a system optimal (SO) perspective. A conditional value-at-risk (CVaR) is incorporated into the objective function to account for risk measures in the presence of uncertain post-disaster demand. We resolve the non-linear travel time function of traffic flow by employing a second-order cone programming (SOCP) approach and linearizing the non-linear chance constraints into a new mixed-integer linear programming (MILP) reformulation so that the problem can be directly solved by state-of-the-art optimization solvers. We illustrate the application of our model using two case studies. The first case study is used to demonstrate the difference between a risk-neutral model and our proposed model. An extensive computational study provides practical insight into the proposed modeling approach using another case study concerning the Black Saturday bushfire in Australia.

## 1. Introduction

Whether they are natural or man-made, disasters such as hurricanes, earthquakes and terrorist attacks inflict serious risk on humankind. In 2010, the Haitian government reported that that an estimated 316,000 people had died, 300,000 had been injured and 1,000,000 were homeless as a result of the Haiti earthquake. This caused a massive level of devastation and imposed enormous operational challenges on the humanitarian agencies and government. Scientific and reasonable shelter assignment and evacuation routing planning are two important safeguards to minimize the loss from disasters.

Different types of disasters require different types of shelter assignments and evacuation planning. For example, earthquakes require emergency evacuation while typhoons allow early evacuation. By means of modern information technology, the relevant government department can analyze and forecast the path and intensity of a typhoon, which can give people sufficient time to evacuate. However, scientists cannot yet calculate when there will be a major earthquake. When an earthquake comes, many people need to evacuate immediately. To promote the efficiency of disasters rescue and reduce life and economic losses, shelter location is significant to the reasonable planning at the pre-disaster phase and evacuation plans are carried out relying upon the information gained (e.g., earthquake magnitude, volcanic eruptive intensity, fire dangerous grade, etc.) when disasters happen. Topics regarding shelter or evacuation, important aspects of disaster relief, have been frequently considered by researchers, and have appeared in emergency supply planning [1,2], shelter location problems [3,4,5], emergency evacuation planning problems [4,5,6,7,8,9,10,11,12,13,14,15,16,17,18,19,20,21,22,23,24,25,26] and shelter location and evacuation routing assignment problems [4,5,8,9,10,11,12,16,27,28].

Shelter location and evacuation routing operations rely on the boundary between disaster preparedness and disaster response. In line with the framework proposed by [4], we assume that shelter location and evacuation routing assignments are disaster response operations.

A shelter is a safe facility that protects people from possible damaging effects of a disaster. It can provide people with necessary living conditions (e.g., food, water, medicine, etc.). People usually start leaving their own houses to seek shelters with a sense of panic when they either face or are experiencing dangerous circumstances. Under those circumstances, people may choose the same path to the shelters. This will reduce the traffic capacity of those roads and delay the time taken to reach the shelter. In addition, space within shelters is limited. Overcrowded shelters cause people who may arrive later to seek a new one. This will further aggravate the condition of people who were wounded by the disaster.

The existing evacuation planning and management models are mostly based on traffic assignment models [29], such as the user equilibrium (UE), the system optimal (SO) and the nearest allocation (NA) approaches. Recently, the Constrained System Optimal (CSO) model [4,5] was developed to provide a compromise between the SO and UE/NA approaches. The UE approach assumes that the evacuees act selfishly and have full information about the traffic conditions on every possible route in the evacuation network. Based on these assumptions, in the UE model, every evacuee can find the optimal route to their destination using an objective function of minimizing their travel time. Clearly, the perfect information assumption in the UE approach is strong and may not be valid in the case of a natural disaster, as the real traffic demand is unusual, and it is difficult for evacuees to guess the congestion level on a path. In the NA model, each evacuee uses the shortest path to reach the nearest open shelter sites, whereas this short-sighted assignment will lead to a poor system performance.

The main goal of evacuation agencies and the governments, however, is to avoid congestion and enable the evacuation of the disaster area in a timely manner. This can be achieved through effective planning strategies, the SO model, which distributes traffic over the road network, and shelter assignment. In the SO model, evacuees are assigned to designated safety and evacuation routes as soon as possible to minimize the total evacuation time. The SO solution may not favor the preferences of some evacuees to the benefit of the other evacuees. For example, some evacuees may be allocated to shelters much further away than they would like to go or to routes much longer than they would normally take.

In this article, we focus on emergency evacuation planning operations for a disaster response, which consists of shelter location decisions and evacuation routing assignments in an SO fashion. In this work, evacuation demand is an uncertain parameter, as shelter location decisions should be made before disasters happen. Contrary to the general expectation-based (i.e., risk-neutral) stochastic model, a risk-averse version that incorporates a risk measure called Conditional Value-at-Risk (CVaR) is established to capture the random parameters to minimize the total evacuation time and associated risk. Considering the utilization balance among shelters in uncertain contexts, on the other hand, chance constraints are introduced to ensure that the utilization level of shelters is not less than a threshold within a given level of reliability.

To summarize, the contribution of the present work is four-fold:

(i) A two-stage stochastic programming shelter location and evacuation routing assignment (SLERA) model with a chance constraint is developed, in which the demand for evacuees is regarded as random. In the first stage, location decisions are made following some possible demand scenarios. The second-stage decision considers specific evacuation routing assignments concerning available shelters as the actual realization of demand.
(ii) Considering the uncertainty of the demand, a risk measure (CVaR) is introduced into the stochastic model to balance the expectations related to the total evacuation time and inherent risk.
(iii) To handle the non-linearity objective function of the traffic flow, a second-order cone programming (SOCP) approach is used.
(iv) Extensive numerical experiments are carried out to provide practical insight into the proposed modeling approach.

The remainder of this work is organized as follows: Section 2 reviews some related work on emergency management, especially related to the disaster response, such as shelter locations and evacuation routing. Expectation-based and risk-averse mathematical models for the SLERA problem are given in Section 3. The solution approach is detailed in Section 4. We present the computational results of the case study in Section 5. Finally, Section 6 concludes this work and indicates some future research directions.

## 2. Literature Review

In this section, we provide a brief introduction to the existing literature, including evacuation planning, shelter location, and an integrated approach of both. Table 1 gives an overview of these works.

### 2.1. Emergency Evacuation Planning Problem

In contrast to conventional emergency evacuation plans that often assign evacuees to fixed routes or shelters, Han et al. [6] proposed a static model that decides on the shelter and route assignment simultaneously by not constraining evacuees to pre-specified shelter sites to ensure the quality of plans while roads are disturbed. In [18], optimal evacuation destination, traffic assignment, and evacuation departure schedule decisions were integrated into a unified optimal traffic flow optimization model.

To represent the time-varying characteristics of the evacuation traffic conditions, dynamic network flow models were employed in the evacuation planning problem [17,20,30]. Specifically, Lim et al. [20] presented a capacity-constrained network flow optimization approach for finding evacuation paths, flows and schedules to maximize the total number of evacuees for short notice evacuation planning against hurricanes. In [17], a conflict-based path-generation approach was proposed for the evacuation planning problem by decomposing the problem into a master and a sub-problem. The sub-problem generates new evacuation paths for each evacuate area, while the master problem optimizes the flow of evacuees and produces an evacuation plan. In [15], the demand management strategies of aggregate-level staging and routing were studied in an attempt to manage large regional evacuations against disaster events by reducing or eliminating congestion in the evacuation network. In [22], the dynamic resource allocation problem was addressed for transportation evacuation planning on large scale networks. In a system optimal dynamic traffic assignment problem, Tuydes-Yaman and Ziliaskopoulos [23] focused on modeling demand management strategies regarding to the optimal departure time, optimal destination choice and optimal evacuation zone scheduling (also known as staggered evacuation) under a given fixed evacuation time assumption.

Most existing evacuation planning problems (i.e., traffic assignment problems) assume that the input parameters, such as evacuation demand and capacity, are deterministic. Notably, some works have accounted for uncertainties in parameters under stochastic settings such as stochastic programming [7], robust optimization [19,24] and chance constraint programming [30,31].

### 2.2. Shelter Site Location Problem

Recently, Mostajabdaveh et al. [14] studied a stochastic scenario-based problem involving the determination of a set of shelter locations in the preparedness stage for natural disasters, which takes into consideration the equity quantified by Gini’s Mean Absolute Difference (called Gini index) under uncertainty. For large-scale problems, a genetic algorithm to solve the sub-problem by mixed-integer programming was proposed.

Motivated by the realization that shelters are shared facilities between the source (relief supply) and demand sides, designing a strategic preparedness network consisting of the evacuation source, shelters and distribution centers [1] naturally integrates the evacuation and relief distribution problem at the strategic and tactical levels simultaneously. Their formulation can make strategic and tactical decisions at the same time before a foreseen disaster, such as a hurricane reaches the area. A benders decomposition approach is used because of the complexity of the integrated model.

### 2.3. Shelter Location and Evacuation Routing Assignment Problem

Within the emergency management context, the shelter location and evacuation routing problems have been studied by numerous optimization researchers, either in a separate or integrated fashion. Bi-level programming, in which shelter location decisions and route assignments are given at the upper and low levels respectively, is one of the most common approaches to address the above two problems separately in the early period [9,10,16,27,28].

Specifically, Kongsomsaksakul et al. [27] studied an optimal shelter location problem for flood evacuation planning with the assumption that the planning authority can control the traffic in certain parts of the evacuation network comprising the evacuation routes of evacuees. Bi-level programming was deployed to model this problem, where the upper lever minimized the total evacuation time and the evacuees’ decisions (e.g., shelter assignment) at the lower level were formulated by a combined distribution and assignment model in line with the shelter location information of the upper level. Similarly, Ng and Waller [16] developed a hybrid bi-level model solved using a simulated annealing algorithm to balance the optimal system and individual components, where the shelter assignment is made first in a system optimal manner and then evacuees are free to choose the optimal route to reach their assigned shelter. Furthermore, bi-level programming was extended to a stochastic context by [10,28] in which a scenario-based shelter location model was proposed to identify a set of robust shelter locations during hurricane events. In [10], user equilibrium-based traffic dynamics were incorporated into the optimization of shelter locations under uncertainty to identify a set of robust shelter locations. The objective of the upper level was to minimize the weighted sum of the expected unmet shelter demand and the expected total travel time of network users. By confining the uncertain demand to an uncertainty set, Kulshrestha et al. [9] presented a robust optimization approach for both shelter location and capacity decisions under demand uncertainty and their proposed model was formulated as a mathematical program with complementarity constraints and was solved using a cutting-plane algorithm.

In order to balance different performance criteria or take into account diverse interest stakeholders, multi-objective models are always formulated [8,11,12,32,33]. A multi-objective shelter location and evacuation routing for an urban evacuation planning problem was proposed by [32], in which primary and secondary routes for evacuees are identified simultaneously. These optimization approaches are embedded into a Geographic Information System-based decision support system so planners can access them via the Internet. In [8], a three-stage multi-objective programming model was used to design a centralized emergency supply network for emergency logistics operations. A shelter network for evacuation was designed in the first-stage phase with a minimized total travel distance.

In contrast to the separate optimization method, the integrated shelter location and evacuation routing problems have received recent attention [4,5,11,12,33,34]. In the realm of emergency evacuation planning under urban area circumstances, multi-criteria optimization models were developed in [11,33]. In particular, Coutinho-Rodrigues et al. [33] introduced a multi-objective location-routing model based on the work proposed by [32] to identify the locations of shelters and evacuation paths for urban evacuation planning. The factors considered in this model are six-fold and include the total travel distance, evacuee risk, total evacuation time, and so on. This model was tested for a simulated fire situation and solved by an off-the-shelf optimization solver. In [11], a comprehensive optimization model based on the multiple commodities flow problem using public and private transport was presented to address the location and routing problems simultaneously. A genetic algorithm based on nondominated sorting genetic algorithm-II (NSGA-II) was designed to solve the multi-objective mixed-integer program, in which the authors modeled the dynamic aspects of an evacuation process and took into account the total evacuation time, risk of the evacuees, and number of shelters to be opened simultaneously. In [4,5], a non-linear mixed-integer model was presented for vehicle-evacuation toward shelter destinations, in which a constrained system optimization (CSO) methodology was introduced to guarantee a level of tolerance so that evacuees are willing to accept the travel routes, even though the assigned routes to shelters are not the shortest ones. The objective of the problem was to minimize the total evacuation time of the entire system, which was modeled through a non-linear function of traffic flow. To handle the non-convex effects of the non-linear objective function, a second-order cone programming (SOCP) method was deployed by transferring the non-linear part of the objective function to a constraint.

Furthermore, Bayram and Yaman [4] extended the CSO shelter location and routing assignment problem based on [5] to a scenario-based stochastic case where the evacuation demand and the potential alterations to the network infrastructure are uncertain before a disaster occurred. The benders decomposition algorithm with diverse cut strategies and the cutting plane algorithm was developed to deal with large-scale problems. In [34], the Turkish Red Crescent (TRC) approach, which is used to address the temporary shelter location and self-evacuation problem, was improved by developing a mathematical model to select the best shelter area locations from a set of criteria. The aim of this model is to determine the best shelter location sites and match evacuation zones to shelter sites while taking into account the utilization of shelters. Focusing on a specific category of support evacuation of late evacuees, in [12], a bi-objective integer programming model was developed to support shelter location and routing decisions during short-notice evacuations. For this model, the support of public authorities is required, and the model is solved by an ϵ-constrained algorithm with two conflicting objectives: maximizing the number of evacuees and minimizing the utilization of resources.

## 3. Problem Description and Model Formulation

**Figure 1:**
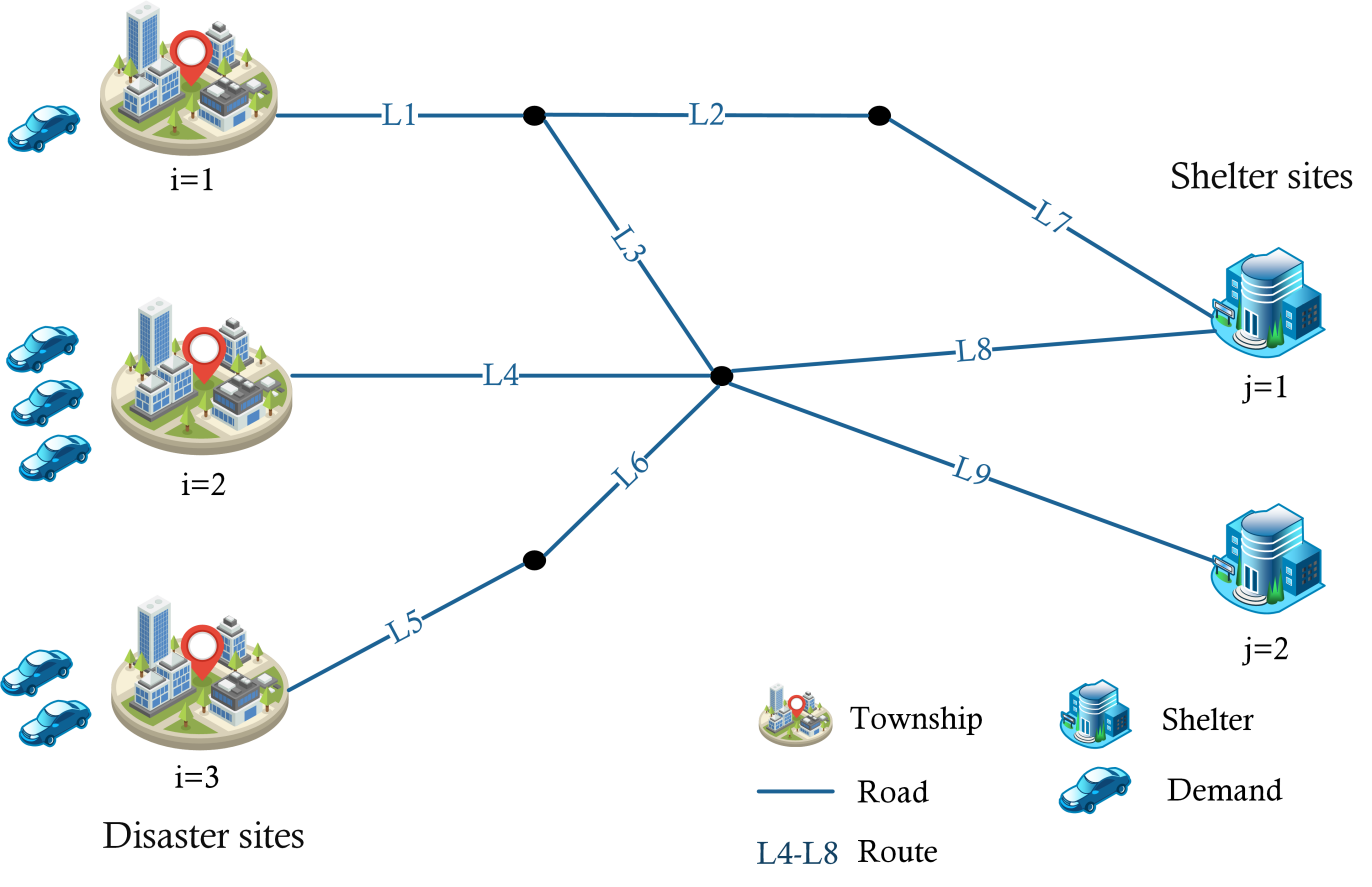
Illustration of the evacuation network.

The aim of our emergency evacuation planning problem is to decide the location of *S* shelters among the potential sites at the tactical level and the routing assignment of evacuees from stricken regions to safe destinations operationally so that the disaster areas are evacuated as quickly as possible. In this work, we focus on evacuation with private vehicles (i.e., car-based evacuation). Figure 1 illustrates the evacuation network. The objective of our problem is to minimize the total evacuation time on all road segments.

Before introducing the mathematical model of the SLERA problem, we first detail how to model the travel time. It is generally known that there is a positive and monotonically increasing relationship between travel time and traffic flow, as a large traffic volume will lead to congestion and increase the travel time on roads. Getting a reasonable estimate of the entire evacuation time is the main reason for modeling traffic time on road segments in accordance with corresponding traffic flows. In the literature, there are different functional forms that model the relationship between travel time and traffic flow on a road segment. In particular, the U.S. Bureau of Public Roads (BPR) function has been extensively adopted in evacuation models to estimate the traffic times, for example, in [4,5]. Thus, the BPR function is applied in this work as well. In BPR function, the travel time *t* is not constant, as the level of congestion has an impact on the travel speed of vehicles, especially during disaster evacuation. According to [5], travel time t(x) is a monotonically increasing function of the traffic flow *x*. The relationship between travel time and traffic flow is formulated as

(1) $$t\left(x\right)=t^{0}\left(1+\gamma {\left(\frac{x}{c}\right)}^{\beta }\right)$$

where t(x) is the travel time when the traffic volume on the road is *x*, *c* denotes the maximum flow capacity, $t^{0}$ is the free-flow travel time at zero volume. The parameters γ≥0 and β≥0 are the tuning coefficients, which determine the shape of the function. The exact values of the coefficients are defined in accordance with the road characteristics, and they are generally taken as 0.15 and 1–5, respectively. When higher flows occur on the link, the coefficient β determines the threshold at which the BPR function rises significantly [35]. Considering that the evacuated tool is the same type of vehicle, a reasonable smaller value of β is preferred. So, we use β=2 in this work.

### 3.1. Notation

The notation used throughout the paper is summarized as follows.

**Sets:**

*I*: The set of demand nodes where the population at risk is to be evacuated, indexed by *i*.
*J*: The set of destination nodes (potential shelter sites) where evacuees reach safety, indexed by *j*.
*R*: The set of all evacuation routes.
$R_{ij}$: The set of alternative routes from demand point i∈I to shelter j∈J, indexed by *r*.
*L*: The set of all road segments in the evacuation network, indexed by *l*.
$R_{l}$: The set of routes passing through road *l*, indexed by *r*.
Ω: The set of possible scenarios in a disaster event, indexed by ω.

**Parameters:**

$d_{i}\left(\omega \right)$: The population of evacuees at demand node i∈I in scenario ω∈Ω.
p(ω): The probability of scenario ω∈Ω.
$c_{l}$: The capacity of road segment l∈L.
$b_{j}$: The capacity of shelter j∈J.
*S*: The number of possible shelters.
$t_{l}^{0}$: The free-flow travel time of road l∈L in zero traffic.
θ: The utilization rate of shelters, where θ∈[0,1].
ϵ: A small value greater than 0.

**Variables:**

$y_{j}$: = 1, if a shelter is located/opened at alternative shelter site j∈J; = 0, otherwise.
$x_{ij}^{r}\left(\omega \right)$: the number of individuals that evacuate from demand node i∈I to shelter j∈J via route r∈R in scenario ω∈Ω.
$f_{l}\left(\omega \right)$: the traffic flow on road l∈L in scenario ω∈Ω.

### 3.2. Expectation-Based Model

Based on the above definition and travel time estimation Equation (1), firstly, the mathematical formulation of the expectation-based problem is written as follows:

(2) $$\left(M1\right)\;\min\;\sum_{\omega \in \Omega }p\left(\omega \right)\sum_{l\in L}t_{l}^{0}\left(1+\gamma {\left(\frac{f_{l}\left(\omega \right)}{c_{l}}\right)}^{\beta }\right)f_{l}\left(\omega \right)$$

s.t.

(3) $$\begin{array}{c} \sum_{j\in J}y_{j}=S \end{array}$$

(4) $$\begin{array}{c} x_{ij}^{r}\left(\omega \right)\leq d_{i}\left(\omega \right)y_{j},\;\forall i\in I,j\in J,r\in R_{ij},\omega \in \Omega \end{array}$$

(5) $$\begin{array}{c} \sum_{i\in I}\sum_{r\in R_{ij}}x_{ij}^{r}\left(\omega \right)\leq b_{j}y_{j},\;\forall j\in J,\omega \in \Omega \end{array}$$

(6) $$\begin{array}{c} P\left(\sum_{i\in I}\sum_{r\in R_{ij}}x_{ij}^{r}\left(\omega \right)\geq \theta b_{j}y_{j}\right)\geq 1-\epsilon ,\;\forall j\in J,\omega \in \Omega \end{array}$$

(7) $$\begin{array}{c} \sum_{j\in J}\sum_{r\in R_{ij}}x_{ij}^{r}\left(\omega \right)=d_{i}\left(\omega \right),\;\forall i\in I,\omega \in \Omega \end{array}$$

(8) $$\begin{array}{c} \sum_{i\in I}\sum_{j\in J}\sum_{r\in R_{ij}\cap R_{l}}x_{ij}^{r}\left(\omega \right)=f_{l}\left(\omega \right),\;\forall l\in L,\omega \in \Omega \end{array}$$

(9) $$\begin{array}{c} y_{j}\in \left\{0,1\right\},\;\forall j\in J \end{array}$$

(10) $$\begin{array}{c} x_{ij}^{r}\left(\omega \right)\in Z^{+}\;\forall i\in I,j\in J,r\in R,\omega \in \Omega \end{array}$$

(11) $$\begin{array}{c} 0\leq f_{l}\left(\omega \right),\;\forall l\in L,\omega \in \Omega . \end{array}$$

The objective Function (2) minimizes the expected total evacuation time spent by the evacuees in the network. Constraint (3) limits the number of open shelters to a pre-specified number *S*. Constraint (4) ensures that evacuees are not assigned to a non-open shelter. Constraint (5) guarantees that the capacity of each opened shelter is not exceeded. The minimum utilization rate of each opened shelter *j* is constrained by chance Constraint (6), which ensures that the utilization rate is not less than a threshold value θ with a specified high probability of 1−ϵ. Constraint (7) ensures that every evacuee from every origin *i* is assigned to a shelter and a route leading to that shelter. The set of Constraint (8) computes the traffic flow on every road segment l∈L. Finally, the domains of the variables are restricted by Constraints (9)–(11).

In M1, the objective criterion is expectation-based (i.e., risk-neutral), and the objective function value entirely depends on the scenario set Ω, which captures the randomness under a finite sample distribution space assumption. The expectation-motivated objective function is reasonable and reliable only if the number of realizations of random demand is large enough. In practice, however, it is difficult to take a large number of scenarios into consideration, due to the restriction of solution techniques. Unavoidably, a case exists where the solutions obtained according to the expectation-based model do not perform well in some extreme demand cases, which does not occur in scenario set Ω. To determine the risk of solutions performing badly in exceptional cases, the introduction of a risk measure method to assess the expectation-based objective value is preferred.

### 3.3. Risk Measure by CVaR

In this subsection, we briefly introduce the concept of CVaR and present a linear programming formulation of CVaR under the assumption that the distribution space of random variables is finite. We recommend readers to refer to [36,37,38] for more details.

As a modeling method, it should be noted that the expectation-based objective Function (2) only accounts for random scenarios in a finite set Ω. The performance of solutions obtained from given scenarios will not be guaranteed under certain realizations of random parameters. To ensure an efficient emergency response operation in which optimal decisions present relatively good stability even in worst-scenarios, especially in the context of disaster management, authors have applied indicators such as the Mean-Variance [39] and CVaR [38] as risk measures. In particular, CVaR has gradually been adopted in pre-disaster relief network design problems [40], emergency logistics planning problems during disasters [2] and disaster location and allocation problems [41]. In these works, risk measure was adopted to avoid a case where the solutions provided by the risk-neutral model might perform poorly in extreme scenarios (e.g., a very high level of unsatisfied demand under certain realizations of random data).

We firstly introduce the concept of the mean-risk model in relation to general two-stage stochastic programming model considering risk aversion. Then, we present the risk measure definition and the risk-averse model, and finally, we conclude this subsection with a linear representation of the risk measure.

(Ω,F,P) denotes an abstract probability space, in which Ω is the sample space, F is a σ-algebra on Ω, and *P* is a probability measure on Ω. In the general risk-neutral two-stage stochastic model, (i.e., expectation-based), the sample space Ω is taken as a finite probability distribution where $\Omega =\{\omega^{1},\ldots ,\omega^{N}\}$. Then, the common expression form of the expectation-based stochastic linear programming problem is written as

(12) $$\min_{x\in X}E\left(f\left(x,\omega \right)\right)=\min_{x\in X}c^{T}x+E\left(Q\left(x,\xi \left(\omega \right)\right)\right)$$

where $f\left(x,\omega \right)=c^{T}x+Q\left(x,\xi \left(\omega \right)\right)$ is the total cost function of the first-stage problem and $Q\left(x,\xi (\omega )\right)$ is the optimal value of the second-stage problem (also known as the recourse function), which is formulated as

(13) $$Q\left(x,\xi (\omega )\right)=\min_{y(\xi )}\left\{q{\left(\xi \right)}^{T}y\left(\xi \right):T\left(\xi \right)x+W\left(\xi \right)y\left(\xi \right)=h\left(\xi \right),y\left(\xi \right)\geq 0\right\}.$$

In the above-mentioned problem, “x” is the first-stage decision variable vector (also referred to as a here-and-now decision), such as the location decision variables in this work, which is made before the realization of the actual random parameter ξ(ω) for the elementary event ω∈Ω; “y” is the second-stage variable vector, like the routing assignment variables, that occurs after a realization of ξ(ω) has been identified. Indeed, this risk-neutral approach that optimizes the cost $c^{T}x$ of the first-stage decision plus the expected cost of the recourse function does not take the risk element into account. The mean-risk model is one of the classical approaches to incorporate the risk measure into a general two-stage stochastic programming model while simultaneously minimizing the risk function. The risk-neutral two-stage framework can be extended to the risk-averse mean-risk version formulated below:

(14) $$\min_{x\in X}\left(1-\lambda \right)E\left[f\left(x,\omega \right)\right]+\lambda \mathrm{CVaR}{}_{\alpha }\left(f\left(x,\omega \right)\right)$$

where λ is a non-negative trade-off coefficient representing the exchange rate between the mean value and risk, called the *risk coefficient* or *risk level*. Specially, the objective Function (14) is risk-neutral when λ=0. For the random variable f(x,ω), CVaR${}_{\alpha }$ denotes the conditional value-at-risk of f(x,ω) at a given confidence level α. The mean-risk model minimizes both the expected cost E(·) and risk measure function CVaR${}_{\alpha }\left(\cdot \right)$. The relevant definitions of the risk measure CVaR are presented as follows:

**Definition** **1.** *Let $F_{Z}\left(\cdot \right)$ represent the cumulative distribution function of a random variable Z. According to [36], the value-at-risk (VaR) at a given confidence level α, denoted by VaR${}_{\alpha }\left(Z\right)$, is defined as*

(15) $$VaR{}_{\alpha }\left(Z\right)=\underset{\eta \in R}{\inf}\left\{F_{Z}\left(\eta \right)\geq \alpha \;\right\}$$

*where α∈(0,1].*

The conditional value-at-risk at a given confidence level α is related to the expectation of *Z* under the condition that it exceeds the α-quantile threshold. Its definition is formulated as

(16) $$\mathrm{CVaR}{}_{\alpha }\left(Z\right)=E(Z|Z\geq VaR_{\alpha }\left(Z\right)).$$

CVaR can capture a wide range of risk preferences by altering another risk coefficient α, in particular, the risk-neutral case (α=0) and the pessimistic worst-case for a sufficiently large value of α→1. *Z* is a random variable that obeys a discretion distribution with finite realizations $\left\{z^{1},\ldots ,z^{N}\right\}$ and corresponding probabilities $\left\{p^{1},\ldots ,p^{N}\right\}$. An alternative definition of CVaR${}_{\alpha }\left(Z\right)$, which gives a linearization description of CVaR, is restated below [36].

**Definition** **2.** *The conditional value-at-risk of random variable Z at the confidence level α is given as*

(17) $$CVaR{}_{\alpha }\left(Z\right)=\underset{\eta \in R}{\inf}\left\{\eta +\frac{1}{1-\alpha }E\left({\left[Z-\eta \right]}_{+}\right)\right\}$$

*where ${\left[Z-\eta \right]}_{+}:=\max\left\{0,Z-\eta \right\}$ and α∈(0,1]. By means of linearizing the term $\left({\left[Z-\eta \right]}_{+}\right)$ in Equation (17), the conditional value-at-risk of random variable Z at the confidence level α can be rewritten as*

(18) $$\begin{array}{cc} CVaR{}_{\alpha }(Z) & =\underset{\eta \in R}{\inf}\left\{\eta +\frac{1}{1-\alpha }E\left(v\right)\right\} \end{array}$$

(19) $$\begin{array}{c} =\min\left\{\eta +\frac{1}{1-\alpha }\sum_{n=1}^{N}p^{n}v^{n}\right\} \end{array}$$

*subject to*

(20) $$\begin{array}{cc} z^{n}-\eta & \leq v^{n},\;\forall n=1,2,\ldots ,N\\ 0 & \leq v,\;\forall n=1,2,\ldots ,N\\ \eta & \in R. \end{array}$$

### 3.4. Risk-Averse Model

Given the definition of risk measure, a mathematical formulation of the stochastic version considering risk measures can be represented as follows. This is based on model M1 and Equations (14) and (18)–(20):

(21) $$\left(M2\right)\;\min\left(1-\lambda \right)\sum_{\omega \in \Omega }p\left(\omega \right)\sum_{l\in L}t_{l}^{0}\left(1+\gamma {\left(\frac{f_{l}\left(\omega \right)}{c_{l}}\right)}^{\beta }\right)f_{l}\left(\omega \right)+\lambda \left(\eta +\frac{1}{1-\alpha }\sum_{\omega \in \Omega }p\left(\omega \right)v\left(\omega \right)\right)$$

subject to

constraints (3)–(11)

(22) $$\begin{array}{c} \sum_{l\in L}t_{l}^{0}\left(1+\gamma {\left(\frac{f_{l}\left(\omega \right)}{c_{l}}\right)}^{\beta }\right)f_{l}\left(\omega \right)-\eta \leq v\left(\omega \right),\;\forall \omega \in \Omega \end{array}$$

(23) $$\begin{array}{c} 0\leq v(\omega ),\;\forall \omega \in \Omega \end{array}$$

(24) $$\begin{array}{c} \eta \in R. \end{array}$$

In this risk-averse model, smaller values of the risk measure part are preferred, as this indicates the corresponding risk value of the solution’s performance loss in exceptional demand cases. λ and α are two important coefficients that are used to tune the risk preferences for decision makers or the uncertainty level of the decision environment. However, we cannot directly solve this risk-averse stochastic model, because the objection function contains a cubic item $f_{l}^{\beta +1}\left(\omega \right)$, which is a nonlinear objective function. Heuristic techniques or the piecewise linear approximation method are frequently used to handle related nonlinear objective functions occurring in mixed-integer programming (MIP) models. Recently, motivated by the advances in second-order cone programming (SOCP), the nonlinear MIP models were reformulated as corresponding second-order conic mixed-integer programs and then directly solved by off-the-shelf solvers (e.g., CPLEX, Gurobi). In the following section, we describe the SOCP solution approach to problems with the nonlinear objective function.

## 4. Solution Approach

In this section, we first give a brief review of second-order cone programming and provide the mixed-integer SOCP formulation of our model. Then, a sample average approximation of the uncertain problem as well as the sample based mixed-integer linear programming (MILP) reformulation of the non-convex chance constraint are presented.

### 4.1. Second-Order Cone Programming Approach

Conic optimization refers to the optimization of a linear function over conic inequalities. With the large number of practical applications and efficient algorithms (e.g., interior algorithms), SOCP has become a state-of-the-art technique used in commercial solvers such as CPLEX. With the availability of commercial solvers, conic integer models have recently started to be used in airline scheduling problem applications [42] and emergency response planning for disasters [4,5].

By employing SOCP, the nonlinearity in the objective function is transferred to the constraint set in the form of second-order quadratic constraints. One of the common uses of conic quadratic inequalities is to represent a hyperbolic inequality, as in the form of Equation (25) (please refer to [43] for more details):

(25) $$u^{2}\leq v_{1}v_{2},\;u,v_{1},v_{2}\geq 0.$$

Then, each hyperbolic inequality can easily be transformed into a second-order cone inequality, as follows:

(26) $$∥2u,v_{1}-v_{2}∥\leq v_{1}+v_{2}.$$

Here, ||·|| denotes the Euclidean norm.

In our work, we define an auxiliary variable $\pi_{l}$ for each l∈L, and the expectation part of the objective function becomes

(27) $$\left(1-\lambda \right)\sum_{\omega \in \Omega }\sum_{l\in L}p\left(\omega \right)\left(t_{l}^{0}f_{l}\left(\omega \right)+\frac{t_{l}^{0}\gamma }{c_{l}^{\beta }}\pi_{l}\left(\omega \right)\right).$$

The road characteristic coefficient β is generally adopted in a range [1, 5], which is explained in Equation (1). In this work, β=2 is adopted to represent $f_{l}^{3}\left(\omega \right)\leq \pi_{l}\left(\omega \right)$ with hyperbolic inequalities of the form

(28) $$\begin{array}{c} f_{l}^{2}\left(\omega \right)\leq m_{l}\left(\omega \right)h_{l}\left(\omega \right),\;\forall l\in L,\omega \in \Omega \end{array}$$

(29) $$\begin{array}{c} m_{l}^{2}\left(\omega \right)\leq \pi_{l(\omega )}f_{l}\left(\omega \right),\;\forall l\in L,\omega \in \Omega \end{array}$$

(30) $$\begin{array}{c} h_{l}=1,m_{l}\left(\omega \right),\pi_{l}\left(\omega \right),f_{l}\left(\omega \right)\geq 0,\;\forall l\in L,\omega \in \Omega \end{array}$$

where $m_{l},\pi_{l},\mu_{l}$ are auxiliary variables that are used to define hyperbolic inequalities. These hyperbolic inequalities are represented by their respective quadratic cone constraints:

(31) $$\begin{array}{c} ∥2f_{l}\left(\omega \right),m_{l}\left(\omega \right)-h_{l}\left(\omega \right)∥\leq m_{l}\left(\omega \right)+h_{l}\left(\omega \right),\;\forall l\in L,\omega \in \Omega \end{array}$$

(32) $$\begin{array}{c} ∥2m_{l}\left(\omega \right),\pi_{l}\left(\omega \right)-f_{l}\left(\omega \right)∥\leq \pi_{l}\left(\omega \right)+f_{l}\left(\omega \right),\;\forall l\in L,\omega \in \Omega \end{array}$$

(33) $$\begin{array}{c} h_{l}\left(\omega \right)=1,m_{l}\left(\omega \right),\pi_{l}\left(\omega \right),f_{l}\left(\omega \right)\geq 0,\;\forall l\in L,\omega \in \Omega . \end{array}$$

Despite the problem still being a nonlinear programming problem in which the nonlinearity element is transferred to the constraint set, it can be solved efficiently when represented as a SOCP. Note that SOCP problems are solved with the barrier algorithm in CPLEX. The resulting conic problem with SOCP constraints is given below:

(34) $$\left(M3\right)\;\min\;\left(1-\lambda \right)\sum_{\omega \in \Omega }\sum_{l\in L}p\left(\omega \right)\left(t_{l}^{0}f_{l}\left(\omega \right)+\frac{t_{l}^{0}\gamma }{c_{l}^{\beta }}\pi_{l}\left(\omega \right)\right)+\lambda \left(\eta +\frac{1}{1-\alpha }\sum_{\omega \in \Omega }p\left(\omega \right)v\left(\omega \right)\right)$$

s.t.

constraints (3)–(11)

(35) $$\begin{array}{c} f_{l}^{{}^{\prime }2}\left(\omega \right)+\rho_{l}^{2}\left(\omega \right)\leq \phi_{l}^{2}\left(\omega \right),\;\forall l\in L,\omega \in \Omega \end{array}$$

(36) $$\begin{array}{c} m_{l}^{{}^{\prime }2}\left(\omega \right)+\tau_{l}^{2}\left(\omega \right)\leq \varphi_{l}^{2}\left(\omega \right),\;\forall l\in L,\omega \in \Omega \end{array}$$

(37) $$\begin{array}{c} 2f_{l}\left(\omega \right)-f_{l}^{{}^{\prime }}\left(\omega \right)=0,\;\forall l\in L,\omega \in \Omega \end{array}$$

(38) $$\begin{array}{c} m_{l}\left(\omega \right)-\rho_{l}\left(\omega \right)=1,\;\forall l\in L,\omega \in \Omega \end{array}$$

(39) $$\begin{array}{c} m_{l}\left(\omega \right)-\phi_{l}\left(\omega \right)=-1,\;\forall l\in L,\omega \in \Omega \end{array}$$

(40) $$\begin{array}{c} 2m_{l}\left(\omega \right)-m_{l}^{{}^{\prime }}\left(\omega \right)=0,\;\forall l\in L,\omega \in \Omega \end{array}$$

(41) $$\begin{array}{c} \pi_{l}\left(\omega \right)-f_{l}\left(\omega \right)-\tau_{l}\left(\omega \right)=0,\;\forall l\in L,\omega \in \Omega \end{array}$$

(42) $$\begin{array}{c} \pi_{l}\left(\omega \right)+f_{l}\left(\omega \right)-\varphi_{l}\left(\omega \right)=0,\;\forall l\in L,\omega \in \Omega \end{array}$$

(43) $$\begin{array}{c} \sum_{l\in L}\left(t_{l}^{0}f_{l}\left(\omega \right)+\frac{t_{l}^{0}\gamma }{c_{l}^{\beta }}\pi_{l}\left(\omega \right)\right)-\eta \leq v\left(\omega \right),\;\forall \omega \in \Omega \end{array}$$

(44) $$\begin{array}{c} 0\leq m_{l}\left(\omega \right),\pi_{l}\left(\omega \right),f_{l}^{{}^{\prime }}\left(\omega \right),m_{l}^{{}^{\prime }}\left(\omega \right),\phi_{l}\left(\omega \right),\varphi_{l}\left(\omega \right),\;\forall l\in L,\omega \in \Omega \end{array}$$

(45) $$\begin{array}{c} \rho_{l}\left(\omega \right),\tau_{l}\left(\omega \right)\in R,\;\forall l\in L \end{array}$$

(46) $$\begin{array}{c} 0\leq v(\omega ),\;\forall \omega \in \Omega \end{array}$$

(47) $$\begin{array}{c} \eta \in R. \end{array}$$

### 4.2. Sample Average Approximate Algorithm and MILP Reformulation of Chance Constraint

The sample average approximate (SAA) algorithm is one of the predominantly used solution methods to tackle random characteristics in stochastic programming. The basic idea of this approach is to replace the original distribution of a random parameter d with a sample Ω of size |Ω|=N, that is, d(ω),ω∈{1,2,…,N}, where *N* is much smaller than the real number of scenarios of d. In SAA, Monte Carlo simulation is used to generate scenarios to represent the demand for evacuees. It is assumed that the demand in a disaster zone is a multivariate uniform distribution U(μ, ∑), where μ and ∑ are the mean and covariance matrix of the random parameter, respectively. |Ω| scenarios are generated after the Monte Carlo simulation has been run, and each scenario has the same probability $\frac{1}{\left|\Omega \right|}$.

After the |Ω| scenarios are generated, the expected objective Function (34) is estimated by the sample average Function (48)

(48) $$\min\;\left(1-\lambda \right)\sum_{\omega \in \Omega }\sum_{l\in L}\frac{1}{\left|\Omega \right|}\left(t_{l}^{0}f_{l}\left(\omega \right)+\frac{t_{l}^{0}\gamma }{c_{l}^{\beta }}\pi_{l}\left(\omega \right)\right)+\lambda \left(\eta +\frac{1}{1-\alpha }\sum_{\omega \in \Omega }\frac{1}{\left|\Omega \right|}v\left(\omega \right)\right).$$

On the other hand, in line with [44], the chance Constraint (6) can be estimated by an indicator function $\frac{1}{\left|\Omega \right|}\sum_{n=1}^{\left|\Omega \right|}I_{\left(0,\infty \right)}\left(\sum_{i\in I}\sum_{r\in R_{ij}}x_{ij}^{r}\left(\omega \right)-\theta b_{j}y_{j}\right)\leq \epsilon$, which requires a certain percentage of the samples to satisfy the chance constraint, where $I_{\left(0,\infty \right)}\left(\cdot \right)$ is an indicator function denoted as

$$I_{\left(0,\infty \right)}\left(x\right)=\left\{\begin{array}{cc} 0, & if\;x\in (0,\infty );\\ 1, & if\;x\notin (0,\infty ). \end{array}\right.$$

The corresponding problem based on the sample average approximate algorithm (refer to as the SAAP) is formulated as follows:

(49) $$(SAAP)\;\min\;\left(1-\lambda \right)\sum_{\omega \in \Omega }\sum_{l\in L}\frac{1}{\left|\Omega \right|}\left(t_{l}^{0}f_{l}\left(\omega \right)+\frac{t_{l}^{0}\gamma }{c_{l}^{\beta }}\pi_{l}\left(\omega \right)\right)+\lambda \left(\eta +\frac{1}{1-\alpha }\sum_{\omega \in \Omega }\frac{1}{\left|\Omega \right|}v\left(\omega \right)\right)$$

s.t.

constraints (3)–(11), (35)–(47).

(50) $$\frac{1}{\left|\Omega \right|}\sum_{n=1}^{\left|\Omega \right|}I_{\left(0,\infty \right)}\left(\sum_{i\in I}\sum_{r\in R_{ij}}x_{ij}^{r}\left(\omega \right)-\theta b_{j}y_{j}\right)\leq \epsilon ,\;\forall j\in J,\omega \in \Omega .$$

Although the chance constraint is converted to a form involving the indicator Function (50) in the SAAP, it is still difficult to be solved directly, as it is not convex. To solve the sample-based chance constraint directly, an equivalent mixed-integer linear programming formulation is deployed to substitute Equation (50) into the SAAP. For a given sample of size |Ω|, auxiliary binary decision variables z(ω),ω∈Ω are introduced. For each scenario ω, z(ω)=0 if the chance constraint is feasible or z(ω)=1, otherwise. Additionally, a big constant number “*M*” is used to model the case when the chance constraint is violated. Therefore, the function of the chance constraint is realized by limiting the sum of binary variable z(ω),ω∈Ω. Finally, the equivalent expression of the chance Constraint (50) is

(51) $$\begin{array}{c} \theta b_{j}y_{j}-\sum_{i\in I}\sum_{r\in R_{ij}}x_{ij}^{r}\left(\omega \right)\leq Mz\left(\omega \right),\;\forall j\in J,\omega \in \Omega \end{array}$$

(52) $$\begin{array}{c} \sum_{\omega =1}^{\left|\Omega \right|}z\left(\omega \right)\leq \left|\Omega \right|\epsilon ,\;\forall \omega \in \Omega \end{array}$$

(53) $$\begin{array}{c} z(\omega )\in \{0,1\},\;\forall \omega \in \Omega . \end{array}$$

Then, the ultimate formulation of the SAA problem can be written as follows:

minSAAP

s.t.

constraints (3)–(11), (35)–(47), (51)–(53).

## 5. Computational Results

In this section, we describe computational experiments that were carried out for two case studies. In the first part of the computational study, we investigate the comparison results with the risk-neutral model to provide insight into our proposed model considering the risk measure. In the second part, we show the effectiveness of the modeling approach and conduct a sensitivity analysis of the risk parameters. In this work, we considered the evacuation pattern as recommended for the movement of victims toward a safe place with the support of vehicles, which should be planned systematically. We coded the proposed model using MATLAB 2018b and solved it using CPLEX solver 12.8 on a DELL PC with a CPU capacity of 4.00 GHz and 32G RAM.

### 5.1. Instance Data

We solved the risk-averse model with different test networks. The test problems we used were from two sources. The first instance, called Sioux Falls, was inherited from [5], which comes from an online library called the Transportation Network Test Problems. Sioux Falls were downloaded from [45], and this instance was used to provide results comparisons and contrasts with the risk-neutral model in the literature [4]. We focused on the second case that was originally developed by [12] to determine the location and evacuation of a bushfire in Australia. In this case study, the region was represented by a network consisting of 11 nodes and 90 links. The detailed information related to instance cases are presented in Appendix A, and the associated codes can be downloaded from the following link: https://pan.baidu.com/s/1QrG2auqDfCbKepnh8EIWOQ.

### 5.2. Comparison of the Results between Risk-Averse and Risk-Neutral Models

Sioux Falls comprises 24 nodes and 76 arches. The details of the network are presented in Appendix A, Table A1. In line with [5], we set nine potential shelter sites (i.e., destination nodes) at nodes 2, 6–8, and 16–20, and the other nodes were sites affected by disaster (i.e., origin/demand nodes). So, there were 135 |O-D| pairs in total. For each |O-D| pair, there were many possible evacuation routes. It was difficult to solve this problem as all available routes were taken into account. Therefore, we chose *K* shortest paths for each |O-D| pair by the Deletion Algorithm [46], which is based on the classical Dijkstra Algorithm. We determined the demand for each origin node randomly between 200 and 600, and we assumed that the maximum traffic flow rate (capacity) of the road segment was 60 vehicles per hour with a free flow speed of 80 km/h in an uninterrupted traffic flow. We considered three types of facility capacity (1000, 2000, and 3000), and the detailed capacity of potential shelter sites was [3000 2000 1000 1000 3000 3000 2000 2000 1000].

In the risk aversion setting, the solution performance under some worst demand scenarios is emphasized so that the obtained solutions are more conservative than those obtained with the risk-neutral setting in the literature [4]. To illustrate this difference, we compared the proposed risk-averse model with the risk-neutral (i.e., expectation based) approach presented in [4]. The experiment setting was S∈{3,4,5,6} and three types of risk coefficients were used to reflect different risk preference levels λ∈{0,0.1,0.5,0.9}. Note that the risk-averse model is transferred into the risk-neutral one when λ=0. Firstly, we took |Ω|=10 as the scenario’s sample size to generate the shelter location decisions for both the risk-averse and risk-neutral models. The corresponding results of the shelter usage rate is reported in Table 2, Table 3 and Table 4. Then, to measure the performance of the obtained solutions in a bigger sample space, we applied the Monte Carlo simulation to generate a bigger sample to test the performance of solutions over a wide range of situations. These simulation results are presented in Table 5. Intuitively, a sound solution not only performs better in a small sample space, but is also guaranteed under general circumstances.

The utilization rate of opened shelters is summarized in Table 2 and Table 3. The column “#shelter” lists the particular opened shelters, and the symbol “-” indicates that the corresponding utilization rate is 0 or the alternative shelter site is not chosen as opened shelter. As shown in Table 2 and Table 3, we can observe that the shelter site with a bigger capacity is preferred when the risk coefficient λ increases such that it exceeds a threshold value. For example, in S=5, as the risk parameter λ increases to 0.1, compared with the location decision of the risk-neutral model, shelter #2, which has a capacity of 3000, is more preferred over shelter #6, which has a capacity of 2000. Moreover, in the risk-neutral model, when λ takes a value of 0.5, shelter site #16, which has a capacity of 3000, is chosen as an open shelter to substitute shelter #17 and λ=0.1. Turning to the objective values presented in Table 4, the total evacuation time of the risk-averse model is greater than that of the risk-neutral one, and it increases without decreasing as the risk coefficient λ increases. In addition, the time spent on evacuation decreases as the number of open shelters increases. However, based on the effect of *S* on the total evacuation time, what can be estimated is that establishing one more shelter adds little to the evacuation of the entire system when the available number of safety facilities is greater than 6. On the other hand, as shown in the right part of Table 4, the CVaR of total evacuation time decreases as λ increases. This means that the risk that the obtained solutions will perform poorly under other unexpected scenarios is relatively reduced.

In light of the location decisions of λ=0.5 and λ=0.9 being identical, we carried out a simulation of cases of “risk-neutral” and “risk-averse” where λ∈{0.1,0.5}. Table 5 presents the simulation results for the total evacuation time under sample sizes of |Ω|∈{50,100,200}. From Table 5, we can observe that the risk-averse model performed slightly better than the risk-neutral one, as the total evacuation time generated by the simulation of the risk-averse approach was less than that of the risk-neutral approach. This rightly correlates with the results of CVaR for the total evacuation time (TET) shown in Table 4, whereby the solution quality is guaranteed in more general scenarios.

### 5.3. Effectiveness and Sensitivity Analysis

In this section, we reference the instance of a bushfire event in Australia that was presented in [12] using their network structure. The Shire of Murrindindi, which experienced a series of bushfires on 7 February 2009, Black Saturday, is located approximately 100 km northeast of Melbourne in Victoria, Australia. It covers an area of 3889 ${\mathrm{km}}^{2}$ and includes the towns of Alexandra, Buxton, Eildon, Flowerdale, Kinglake, Marysville, Molesworth, Strath Creek, Taggerty, Yarck, and Yea. The study area consists of six main townships which suffer from fire risk: Narbethong $i_{1}$, Marysville $i_{2}$, Taggerty $i_{3}$, Buxton $i_{4}$, Cambarville $i_{5}$, and Rubicon $i_{6}$. In [12], the demand of evacuees was regarded as deterministic, as given in Table 6. Conversely, we assumed that the actual demands in terms of disaster zones were random and independent. We used the deterministic demand reported in Table 6 as the expectation *d* of uncertain demand.

**Figure A2:**
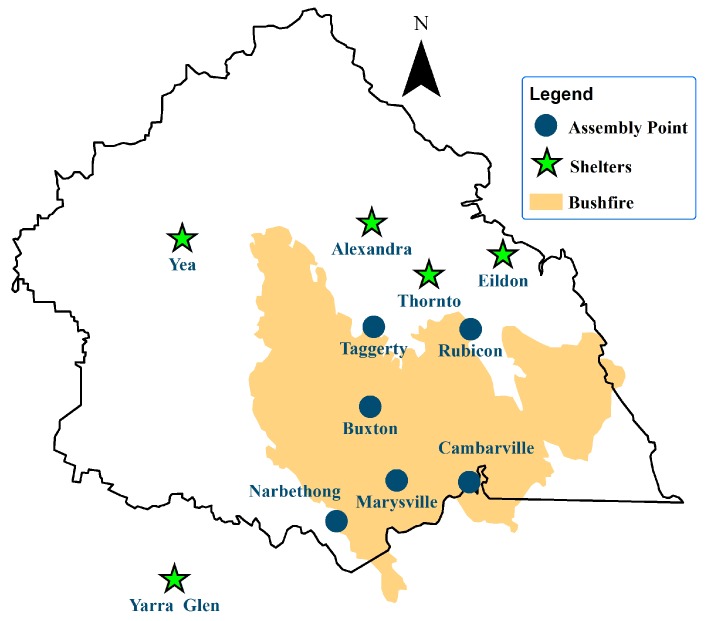
Case study 2—Murrindindi Shire, Victoria, Australia

Five shelters in adjacent townships (destinations) were nominated by Country Fire Authority (CFA): Goughs Bay Fire Station in Alexandra $j_{1}$, a recreation reserve oval in Thornton $j_{2}$, a basketball court in Eildon $j_{3}$, a skate park in Yea $j_{4}$, and a racecourse track in Yarra Glen $j_{5}$. Figure A2 in Appendix A gives the location distribution of these disaster regions and safe destinations. Each shelter has a maximum capacity for evacuees; these capacity values are reported in Table 6. The details of the road segments and road network information are provided in Appendix A, Table A1 and Table A2. We set the capacity for each road segment randomly with a maximum traffic flow rate of 30 vehicles per minute and a free flow speed of 100 km/h. To balance the utilization rates among opened shelters, we set θ=0.2, and the reliability level was ϵ=0.1. Note that despite a much smaller value of ϵ than 0.1 being desirable, we chose ϵ=0.1 as a result of the limited solution ability in this work. Specifically, the reliable level ϵ depends on the sample size of |Ω| in the sample-based chance Constraint (50), so the larger |Ω| is, the smaller ϵ can be, for example, ϵ=0.05 was feasible since the sample size was |Ω|>20.

#### 5.3.1. Effectiveness Analysis

To illustrate the validity of our proposed model, it is necessary to compare the output of computation results with real evacuation outcome. However, it is difficult to obtain the real evacuation schemes used in the bushfire disaster event, as the features of evacuation activity were real-time and spontaneous. In general emergency evacuation events, the nearest allocation (NA) fashion is mostly used to assign shelters and corresponding paths to evacuees, as the information on path lengths or free-flow travel time is more accessible to evacuees compared with actual travel times in a disaster [4]. On the other hand, in an emergency or chaotic environment, it is easy for evacuation personnel to self-select the shortest route based on the distance/travel time to reach a safe destination as quick as possible. Therefore, we used the calculation results generated by the NA approach to approximate what happened in real evacuation case and compared the computational results between our model and the NA method to test the validity of our model.

We assumed that demand distribution of origin nodes was uniform with the means given in Table 6, and the distribution interval was [(1−e)d,(1+e)d] where 0<e<1. Taking e∈{0.1,0.3,0.5} to capture different demand fluctuations, we randomly generated 4 scenarios for each distribution pattern with different numbers of opened shelters S∈{2,3,4,5}. The set of total evacuation times is summarized in Table 7. We can make several observations from the table:

(1) Our proposed model outperforms the NA approach for real evacuation case, as the total evacuation time in our model is far less than that of the NA. Specifically, the maximum and minimum values of the total evacuation time generated by our model are 233,274 and 63,634, respectively. In contrast, the associated values of the NA method are, respectively, 309,844 and 2,612,969.
(2) In terms of the number of opened shelters, the total evacuation time of evacuees decreases as the number of available open shelters increases. Moreover, the contribution of adding one more opened shelter to the reduction in evacuation time shows a marginal diminishing trend in our model. Furthermore, the total evacuation time under the NA case conversely increases as the *S* exceeds 4, which means the open shelters exceed redundancy and a reasonable *S* exists.
(3) In regard to the influence of demand fluctuation, we can see that except for scenario #2, the larger *e* is, the shorter the evacuation time required is.

**Figure 2:**
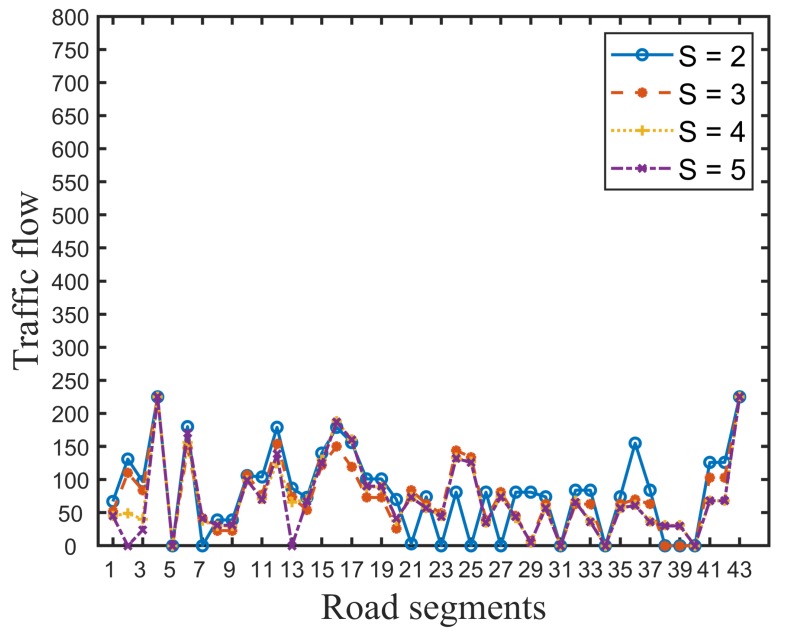
Traffic flow of the road segments using the proposed model.

**Figure 3:**
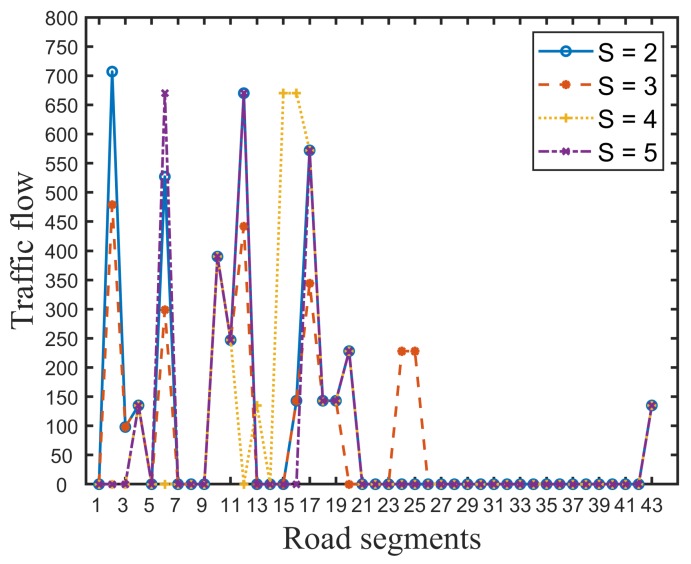
Traffic flow of the road segments using the NA approach.

In order to gain insight into the performance difference, Figure 2 and Figure 3 presents the traffic flow distribution in the evacuation process. Compared with Figure 2 and Figure 3, we can see that the traffic flow in the evacuation paths generated by our model is evenly distributed, and the upper bound of flow in each road segment is less than 250. However, in the traffic flow distribution of NA, road segments 2, 6, 10, 12, and 17 are the main converging roads to safe destinations and road segments 27–42 are idle. Certainly, compared with Figure 2, the probability and severity of traffic congestion in the real evacuation case on road segments 2, 6, 10, 12, and 17 is large, as the traffic flow exceeds the road capacity. The unbalanced distribution of traffic flow directly results in poor system efficiency during emergency evacuations.

#### 5.3.2. The Impact of Risk Coefficients on CVaR and the Total Evacuation Time

In the risk-averse shelter location and routing assignment problem, the parameter λ and the confidence level α are two key coefficients related to shelter location decisions and emergency evacuation planning. For example, a higher λ indicates that more emphasis is placed on the risk part and the corresponding decisions tend to be more conservative to avoid cases of extreme demand. In this part of the computational study, we analyzed the results based on the bushfire evacuation problem with |Ω|=10 and the risk parameters λ∈{0.1,0.2,0.3,0.4,0.5,0.6,0.7,0.8,0.9}, α= {0.6, 0.7, 0.8, 0.9, 0.95, 0.99} under different numbers of available shelters S={1,2,3,4,5}. We assumed that the uncertain demand of evacuees is a uniform distribution of [(1−e)d,(1+e)d], where e=0.3.

**Figure 4:**
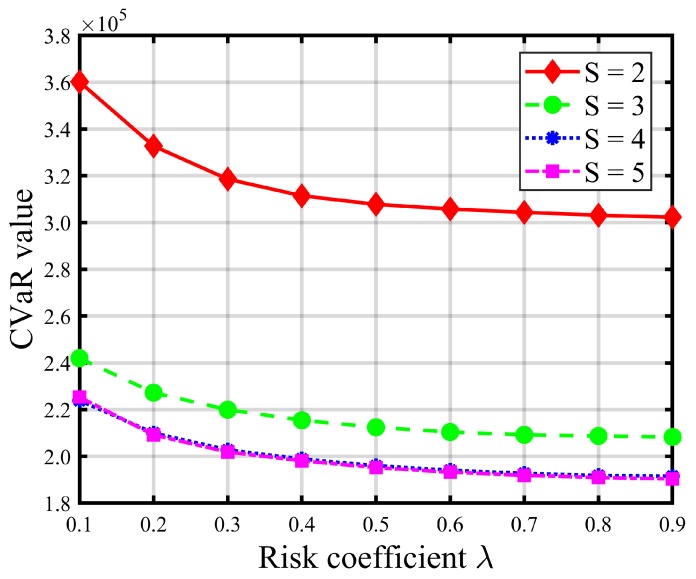
CVaR under different risk coefficients λ (α=0.95).

**Figure 5:**
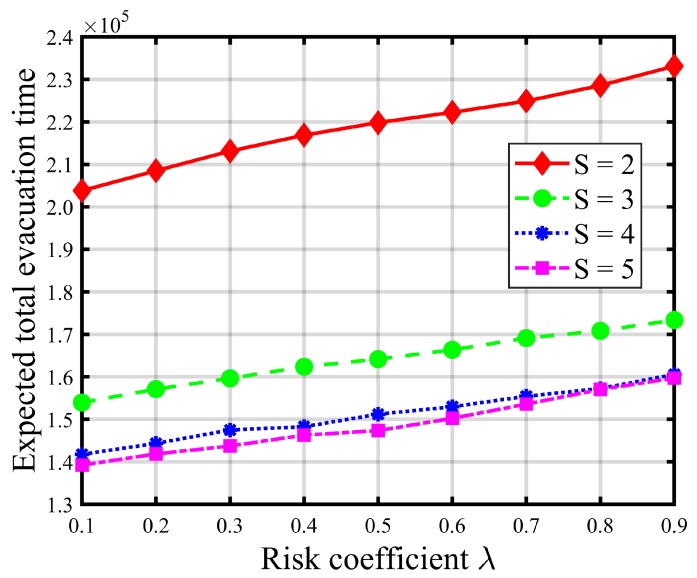
Total evacuation time under different risk coefficients λ (α=0.95).

**Figure 6:**
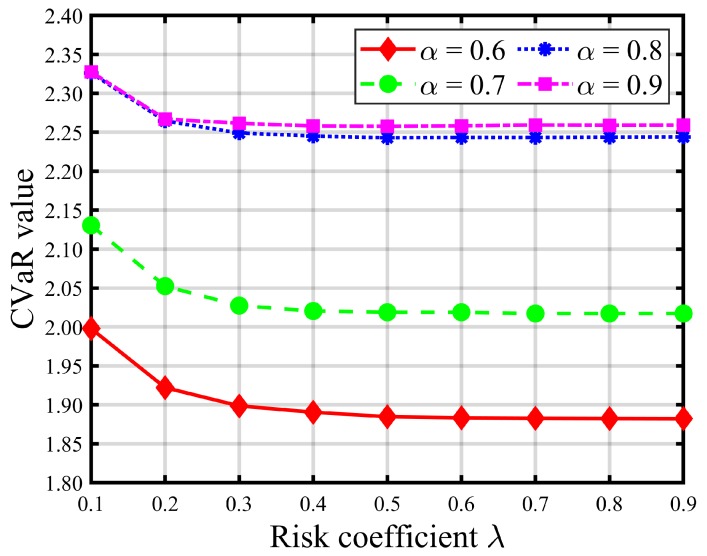
CVaR value of the total evacuation time with different risk coefficients λ (S=1).

**Figure 7:**
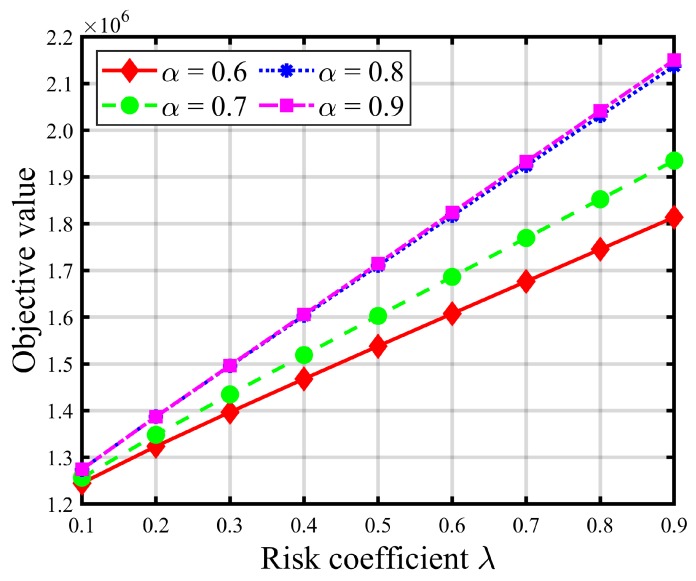
Objective value with different risk coefficients λ (S=1).

**Figure 8:**
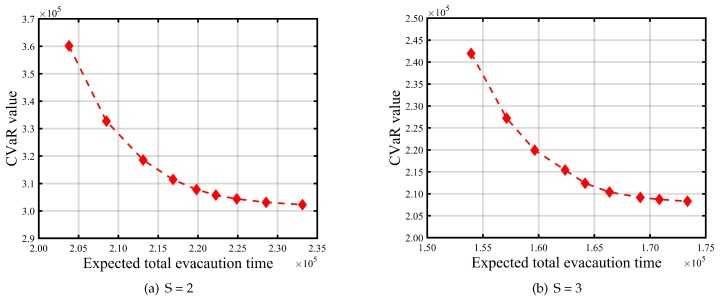
Expected total evacuation time vs. CVaR value with λ∈{0.1,0.2,…,0.9} and α=0.95.

Table 8 and Table 9 report the impacts of risk parameters on the expected total evacuation time and CVaR. Figure 4 and Figure 5 give graphic illustrations of the impacts of λ on the trade-off between the expected total evacuation time (TET) and the associated CVaR under different available shelter cases. Figure 6 and Figure 7 show the impacts of the confidence level α on the risk and objective function value. Figure 8 represents an efficient frontier of risk and expectation.

In the risk-averse model, higher values of λ and α indicate a more conservative decision preference and suggest that more attention should be paid to some extreme realizations of uncertain demand to avoid loss of performance in the solutions. Thus, increasing risk parameter λ brings about more weight to the risk measure value (i.e., CVaR) of the total evacuation time at the expense of increasing the expected TET. As shown in Figure 4 and Figure 5 (as well as Table 8 and Table 9), the CVaR of TET decreases and gradually converges to a fixed value as λ increases, while the expectation of TET increases. These increments, shown in Figure 5 and Table 8, become more pronounced when the number of opened shelters *S* is small, such as S≤3. In addition, we can also observe that the graphs of the expected TET and corresponding CVaR of larger *S* values are always below those of the smaller *S* values, which implies that improvements in CVaR, and thus the level of compromise from the expectation, increase with *S*. Besides, it is obvious that increasing the number of opened shelters will not promote a significant decrease in the total evacuation time once *S* is greater than 3. In contrast, it is resource-wasteful.

Turning to Figure 6, we can observe that a higher α value leads to a greater CVaR, which illustrates that CVaR is a non-decreasing function of α. Similarly, the same relationship is valid for the objective function value graphs in Figure 7. This consequence can be explained by the definition of CVaR in which a smaller confidence level α indicates a worse realization. According to both figures, we can say that the influence of the risk coefficient λ becomes more significant as larger α values are given. Figure 6 and Figure 7 further confirm that the contribution to improving the emergency evacuation effect of simply increasing the number of shelters is small when *S* exceeds 4.

Furthermore, to facilitate the decision makers who are seeking to reach the right balance between expectation of TET and accompanying risk, Figure 8 lists solutions to the expected TET and corresponding risk with different *S* values when α=0.95. As shown in Figure 8, along with the left tails of curves (starting from smaller λ values), the CVaR of TET decreases significantly at the cost of increasing the expected TET. On the other hand, it is obvious that the right tails of these curves tend to be flat. This means that higher compromises from the expected TET are required, especially in the case where the risk coefficient λ is close to 1, to further decrease the risk.

## 6. Conclusions

In this work, we studied a shelter location and routing assignment problem under random demand circumstances and introduced a risk-averse stochastic programming optimization model. Our aim was to minimize the total evacuation time using a system optimal style. To handle the non-linearity in the objective value arising from using a traffic assignment model, second-order cone programming was deployed to transfer the non-linearity from the objective function to constraints. In addition, we performed a series of numerical analyses to validate our proposed model and the impacts of key features were discussed for decision making purposes. This emergency management strategy involving risk measure is valuable as it could support plan making on both tactical and operational levels.

There are some further research improvements that could be made. For example, we adopted a system optimal method, in which we assumed that evacuees would comply with the guidelines released by the central authority. In practice, however, the system optimal routing assignment results may be unfavorable to some evacuees, and people’s behaviors in unusual circumstances are ignored. Thus, the incorporation of human behaviors into the optimization model deserves investigation. On the other hand, more scenarios used to describe randomness for which the use of efficient solution algorithms (both exact and heuristic) to handle large-scale scenarios should be captured. 

## Figures and Tables

**Table 1:** Overview of the literature review.

Author	Traf. Assign.	Shel. Utili.	Uncertainty	Risk-Averse	Route Assign.	Shel. loc.	Solution Method
[6]	SO	×	×	×	*√*	×	Simulation-based
[18]	SO	×	×	×	*√*	×	Commercial solver
[20]	SO	×	×	×	*√*	×	Dijkstra’s algo, greedy algo
[17]	SO	×	×	×	*√*	×	Column generation
[15]	SO	×	×	×	*√*	×	Exact, heuristic
[21]	SO	×	×	×	*√*	×	Exact
[22]	SO	×	×	×	*√*	×	Benders decomposition
[23]	SO	×	×	×	*√*	×	Tabu search
[19]	SO	×	*√*	×	*√*	×	Ant colony algo
[16]	SO	×	*√*	×	*√*	×	Commercial solver
[25]	SO	×	*√*	×	*√*	×	Sample average approximation
[3]	×	*√*	*√*	×	×	*√*	Commercial solver
[27]	SO; UE	×	×	×	*√*	*√*	Genetic algo
[8]	×	×	×	×	*√*	*√*	Commercial solver
[5]	CSO	×	×	×	*√*	*√*	SOCP, commercial solver
[9]	UE	×	*√*	×	×	*√*	Cutting plane algo
[10]	SO; UE	×	*√*	×	*√*	*√*	Lagrangian relax
[11]	SO	×	×	×	*√*	*√*	NSGA-ii@
[12]	×	×	×	×	*√*	*√*	ϵ-constrained algo
[4]	CSO	×	*√*	×	*√*	*√*	SOCP, Benders decomposition
[14]	×	×	*√*	×	×	*√*	Genetic algo
This work	SO	*√*	*√*	*√*	*√*	*√*	SOCP, approximation

**Table 2:** Utilization rate of open shelters under risk-neutral and risk aversion settings (S∈{3,4}).

S=3					S=4				
#Shelter	Risk-Neutral	Risk Aversion			#Shelter	Risk-Neutral	Risk Aversion		
		λ=0.1	λ=0.5	λ=0.9			λ=0.1	λ=0.5	λ=0.9
2	-	-	62.1%	61.8%	2	-	-	59.7%	59.7%
6	99.3%	98.8%	-	-	6	98.2%	97.6%	-	-
16	79.8%	78.8%	80.8%	80.8%	16	61.8%	61.5%	64.8%	64.5%
19	75.4%	77.2%	80.0%	80.3%	19	53.3%	54.4%	58.0%	59.0%
-	-	-	-	-	20	100.0%	100.0%	98.9%	98.1%

**Table 3:** Utilization rate of open shelters under risk-neutral and risk aversion settings (S∈{5,6}).

S=5					S=6				
#Shelter	Risk-Neutral	Risk Aversion			#Shelter	Risk-Neutral	Risk Aversion		
		λ=0.1	λ=0.5	λ=0.9			λ=0.1	λ=0.5	λ=0.9
2	-	50.1%	49.1%	48.4%	2	-	49.2%	49.3%	49.1%
6	79.6%	-	-	-	6	77.3%	-	-	-
-	-	-	-	-	7	46.1%	46.6%	45.3%	45.2%
8	86.6%	88.4%	86.4%	83.1%	8	58.2%	66.9%	62.3%	62.1%
16	-	-	50.9%	51.0%	16	-	-	49.2%	49.3%
17	50.0%	50.9%	-	-	17	49.2%	49.3%	-	-
19	49.2%	51.7%	53.2%	56.2%	19	48.8%	48.8%	51.1%	51.6%
20	94.3%	93.7%	95.8%	94.8%	20	84.5%	82.2%	83.4%	83.5%

**Table 4:** Total evacuation time and corresponding Conditional Value-at-Risk (CVaR).

S	Expected Total Evacuation Time (TET)				CVaR of TET			
	Risk-Neutral	Risk Aversion			Risk-Neutral	Risk Aversion		
		λ=0.1	λ=0.5	λ=0.9		λ=0.1	λ=0.5	λ=0.9
3	6,630,398	6,677,721	7,188,617	7,449,398	-	8,822,657	7,852,525	7,744,518
4	5,963,488	6,010,120	6,491,566	6,720,137	-	7,374,074	6,504,996	6,414,576
5	5,720,957	5,753,614	6,025,619	6,314,789	-	6,927,350	6,228,670	6,197,845
6	5,677,441	5,752,044	5,960,513	6,238,642	-	6,641,029	5,967,101	5,901,922

**Table 5:** Monte Carlo simulation of the evacuation time.

S	|Ω|=50			|Ω|=100			|Ω|=200		
	Risk-Neutral	Risk Aversion		Risk-Neutral	Risk Aversion		Risk-Neutral	Risk Aversion	
		λ=0.1	λ=0.5		λ=0.1	λ=0.5		λ=0.1	λ=0.5
3	6,844,813	6,844,813	6,750,086	6,823,550	6,823,550	6,664,941	6,868,368	6,868,368	6,757,147
4	6,185,932	6,185,932	5,812,245	6,249,512	6,249,512	6,051,388	6,100,323	6,100,323	6,074,580
5	5,758,636	5,626,734	5,605,648	5,932,800	5,835,622	5,783,792	5,870,288	5,828,749	5,717,690
6	5,798,878	5,669,183	5,489,375	5,834,511	5,833,913	5,800,919	5,814,711	5,725,453	5,777,178

**Table 6:** The case study demand and capacity data.

Index *i*	Evacuation Nodes	Demand ($d_{i}$)	Index *j*	Shelters	Capacity ($b_{j}$)
1	Narbethong	240	1	Yea	1500
2	Marysville	260	2	Alexandra	500
3	Taggerty	170	3	Thornton	500
4	Buxton	130	4	Eildon	1000
5	Cambarville	110	5	Yarra Glen	1000
6	Rubicon	190			

**Table 7:** Computation results of the model and real evacuation case.

*e*	*S*	Scenario #1		Scenario #2		Scenario #3		Scenario #4	
		Model	NA	Model	NA	Model	NA	Model	NA
0.1	2	197,562	1,675,112	214,904	1,890,489	181,986	1,451,272	207,581	1,711,341
	3	150,207	713,215	162,734	769,519	138,477	653,297	157,078	754,420
	4	132,548	877,519	142,299	984,507	122,061	789,196	137,270	922,060
	5	129,972	952,549	139,549	1,076,953	119,591	854,861	134,562	1,002,812
0.3	2	171,134	1,517,710	222,062	2,207,604	130,294	942,740	196,157	1,604,709
	3	133,512	618,941	170,368	806,720	102,109	459,105	150,727	721,702
	4	119,562	719,198	148,606	1,053,067	90,991	501,065	131,155	829,372
	5	117,286	776,878	145,842	1,162,791	88,993	538,252	128,529	903,243
0.5	2	149,986	1,392,877	233,274	2,612,968	89,529	565,754	186,844	1,508,880
	3	120,224	552,166	181,197	891,254	72,416	308,340	145,627	700,811
	4	109,426	593,075	157,991	1,172,871	65,200	292,294	126,431	746,900
	5	107,425	634,276	155,209	1,301,356	63,634	309,844	123,790	813,580

**Table 8:** Expected total evacuation time.

*S*	α	λ								
		0.10	0.20	0.30	0.40	0.50	0.60	0.70	0.80	0.90
2	0.60	222,277	226,809	213,374	216,567	219,922	223,513	225,850	225,705	231,960
	0.70	203,786	208,836	213,434	216,737	219,640	222,360	225,387	228,950	232,450
	0.80	203,799	208,495	213,124	216,875	219,852	222,283	224,977	228,695	232,920
	0.90	203,784	208,443	213,121	216,877	219,824	222,245	224,890	228,565	233,150
	0.95	203,802	208,499	213,124	216,875	219,826	222,248	224,890	228,565	233,140
	0.99	203,788	208,486	213,124	216,877	219,824	222,245	224,893	228,565	233,140
3	0.60	155,109	156,859	159,541	162,675	165,168	166,470	168,940	170,150	172,230
	0.70	154,796	156,328	160,073	161,607	164,670	167,555	168,590	170,635	173,040
	0.80	154,848	157,061	159,374	162,102	164,814	167,263	168,843	170,385	173,300
	0.90	154,297	156,110	158,689	162,370	163,952	167,258	168,847	170,565	172,310
	0.95	153,946	157,124	159,649	162,375	164,168	166,360	169,127	170,835	173,360
	0.99	155,089	156,598	160,019	162,375	164,766	166,393	169,127	170,525	173,050
4	0.60	142,463	144,243	145,770	147,507	151,934	152,878	156,070	159,135	160,860
	0.70	142,189	144,325	146,656	148,263	149,748	153,478	155,467	157,170	160,150
	0.80	142,893	144,391	146,811	147,492	150,618	153,790	154,840	158,525	161,000
	0.90	142,987	143,008	144,779	148,577	151,074	150,250	154,453	158,800	159,810
	0.95	141,773	144,334	147,500	148,263	151,216	152,960	155,437	157,250	160,580
	0.99	141,553	144,849	145,374	147,252	149,890	152,505	154,627	157,250	160,580
5	0.60	136,120	141,029	142,881	146,360	147,220	149,663	153,127	155,405	158,300
	0.70	139,809	143,095	143,404	146,298	146,858	151,343	152,813	155,005	159,730
	0.80	136,463	142,401	143,231	146,298	149,464	149,748	153,590	157,050	159,730
	0.90	136,906	143,096	142,211	147,120	148,138	150,555	154,250	156,410	159,730
	0.95	139,217	141,880	143,717	146,298	147,346	150,240	153,587	157,050	159,730
	0.99	137,277	143,096	142,923	145,507	148,676	150,248	153,767	157,050	159,730

**Table 9:** Conditional value-at-risk (CVaR) of the total evacuation time.

*S*	α	λ								
		0.10	0.20	0.30	0.40	0.50	0.60	0.70	0.80	0.90
2	0.60	409,540	382,085	313,290	307,288	303,164	300,223	298,931	297,724	301,001
	0.70	360,180	330,580	316,367	310,113	306,534	304,287	302,666	301,455	300,832
	0.80	360,190	332,660	318,560	311,450	307,742	305,738	304,297	303,039	302,288
	0.90	360,140	332,575	318,567	311,448	307,784	305,787	304,370	303,129	302,318
	0.95	360,190	332,725	318,560	311,450	307,782	305,785	304,370	303,133	302,274
	0.99	360,140	332,685	318,560	311,448	307,784	305,787	304,367	303,119	302,308
3	0.60	241,560	225,195	218,010	213,398	210,322	208,533	207,479	207,081	206,684
	0.70	241,970	234,500	219,293	214,740	211,562	209,538	208,470	208,073	207,678
	0.80	241,980	226,930	219,940	215,423	212,414	210,402	209,187	208,725	208,304
	0.90	250,160	238,245	232,963	215,423	212,414	210,402	209,189	208,725	208,306
	0.95	241,980	227,235	219,940	215,423	212,414	210,402	209,187	208,726	208,304
	0.99	241,980	226,930	219,807	215,423	212,414	210,402	209,187	208,725	208,304
4	0.60	238,290	209,915	202,370	198,495	195,640	193,680	192,359	191,561	191,169
	0.70	243,200	210,025	202,883	198,845	196,108	194,068	192,716	191,864	191,451
	0.80	241,510	208,740	202,760	198,845	196,108	194,068	192,707	191,746	191,211
	0.90	224,350	209,990	202,763	198,843	196,108	194,138	192,716	191,866	191,451
	0.95	223,900	209,990	202,763	198,845	195,994	194,070	192,707	191,865	191,450
	0.99	240,950	208,740	202,760	198,843	195,994	194,068	192,716	191,865	191,450
5	0.60	225,070	207,265	201,503	197,433	194,748	192,742	191,329	190,388	189,821
	0.70	223,040	207,820	202,070	198,110	195,100	193,158	191,811	190,860	190,300
	0.80	242,500	207,765	201,853	198,110	195,102	193,190	191,863	190,926	190,343
	0.90	225,390	207,815	201,853	197,985	195,100	193,238	191,859	190,919	190,343
	0.95	225,380	209,165	201,853	198,110	195,186	193,243	191,864	190,926	190,343
	0.99	223,020	207,815	201,853	198,110	195,186	193,238	191,859	190,926	190,343

**Figure A1:**
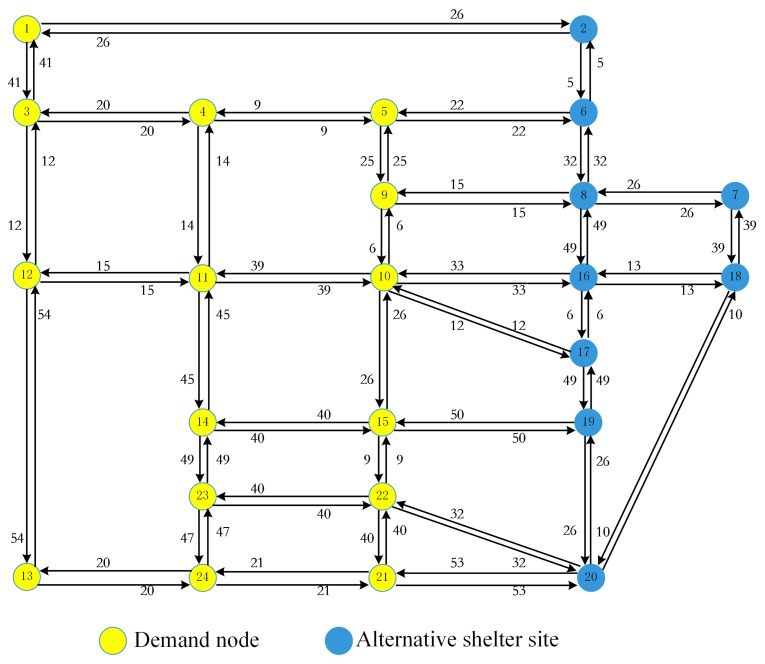
Case study 1—Sioux Falls network.

## Appendix A

**Table A1:** Road segment data from case study 2.

Code	Road Name	Travel Time	Code	Road Name	Travel Time
		(min)			(min)
L1	Eildon-Warburton, Eildon Jamieson Rd	132	L23	Stony Creek	75
L2	Goulburn Valley Hwy (B340)	11	L24	Maroondah Hwy (B360)	23
L3	Back Eildon Rd	11	L25	Healesville-Yarra Glen Rd (C726)	13
L4	Rubicon Rd	12	L26	Healesville-Kinglake Rd (C724)	17
L5	Blue Range Rd	16	L27	Melba Hwy (B300)	14
L6	Taggerty-Thornton Rd (C515)	4	L28	Healesville-Kinglake Rd (C724)	7
L7	Bulls Ln	10	L29	Melba Hwy (B300)	13
L8	Blue Range Rd	52	L30	Murrindindi Rd	66
L9	Lake Mountain-Royston River Rd	68	L31	Black Range Rd	70
L10	Marysville-Woods Point Rd (C512)	19	L32	Cameron Rd	55
L11	Mount Margaret Rd	49	L33	Simmonds Track	20
L12	Taggerty-Thornton Rd (C515)	6	L34	Myles Rd	15
L13	Goulburn Valley Hwy (B340)	11	L35	Murrindindi Rd	7
L14	Breakaway Rd-Hoban Rd	12	L36	Melba Hwy (B300)	14
L15	Maroondah Hwy (B360)	8	L37	Limestone-Ginter Rd	53
L16	Maroondah Hwy (B360)	6	L38	Scrubby-Black Range Rd	45
L17	Maroondah Hwy (B360)	8	L39	Whanregarwen Rd	12
L18	Buxton-Marysville Rd (C508)	3	L40	Whanregarwen Rd	11
L19	Buxton-Marysville Rd (C508)	9	L41	Goulburn Valley Hwy (B340)	13
L20	Maroondah Hwy (B360)	12	L42	Goulburn Valley Hwy (B340)	10
L21	Marysville Rd (C512)	10	L43	Rubicon Rd	3
L22	Plantation Rd	25			

**Table A2:** Network information for case study 2.

From	To	Route 1			Route 2			Route 3		
		Road Segments	Travel Time	Distance	Road Segments	Travel Time	Distance	Road Segments	Travel Time	Distance
			(min)	(km)		(min)	(km)		(min)	(km)
Narbethong	Yea	L24-L26-L28-L29-L36	74	85	L22-L30-L35-L36	112	63	L20-L17-L14-L41-L42	63	69.4
	Alexandra	L20-L17-L16-L15	34	47	L21-L19-L18-L17-L16-L14	48	52	L20-L17-L12-L6-L13	41	50.7
	Thornton	L20-L17-L12-L6	30	38	L21-L19-L18-L17-L12-L6	40	45	L21-L19-L11-L8-L7-L6	134	65.1
	Eildon	L20-L17-L12-L6-L2	41	52	L21-L19-L18-L17-L12-L6-L2	51	58	L21-L10-L1	161	90.5
	Yarra	L24-L25	36	35	L24-L26-L28-L27	61	62	L23-L7-L27	99	42.9
Marysville	Yea	L19-L18-L17-L16-L14-L39-L40-L42	71	71	L19-L18-L17-L16-L15-L41-L42	57	73	L19-L18-L32-L33-L37	140	69.2
	Alexandra	L19-L18-L17-L16-L15	34	42	L19-L18-L17-L12-L6-L13	41	45	L19-L11-L8-L7-L6-L13	135	65.5
	Thornton	L19-L18-L17-L12-L6	30	33	L19-L11-L8-L7-L6	124	53	L19-L18-L17-L16-L14-L13	49	52.8
	Eildon	L19-L18L17-L12-L6-L2	41	46	L10-L1	19	17	L19-L11-L8-L7-L6-L2	135	66.5
	Yarra	L21-L24-L25	46	47	L21-L24-L26-L28-L27	71	74	L21-L23-L28-L27	106	58.8
Buxton	Yea	L17-L16-L14-L39-L40-L42	59	59	L16-L15-L41-L42	37	49	L32-L33-L37	75	34.4
	Alexandra	L17-L16-L15	22	30	L17-L12-L6-L13	29	33	L32-L38-L39	112	52.3
	Thornton	L17-L12-L6	18	21	L17-L16-L15-L13	33	42	L18-L11-L8-L10-L6	127	62.3
	Eildon	L17-L12-L6-L2	29	34	L17-L16-L14-L13-L2	48	54.4	-	-	-
	Yarra	L20-L24-L25	48	53	L19-L21-L24-L25	67	66	L20-L23-L28-L27	108	64.1
Taggerty	Yea	L16-L15-L41-L42	37	49	L16-L14-L39-L40-L42	51	48	L17-L32-L33-L37	136	69
	Alexandra	L16-L15	14	18	L16-L14	18	17	L12-L6-L13	21	21.8
	Thornton	L12-L6	10	9.4	L16-L15-L13	25	31	L16-L14-L13	29	29.4
	Eildon	L12-L6-L3	21	21	L12-L6-L2	21	23	L16-L15-L13-L2	36	43.9
	Yarra	L17-L20-L24-L25	56	64	L17-L20-L24-L28-L27	78	67.2	L16-L15-L42-L41-L36-L29-L27	78	107
Cambarville	Yea	L10-L19-L18-L17-L16-L15-L41-L42	76	89	L9-L8-L7-L12-L16-L15-L41-L42	163	101	L10-L19-L18-L23-L33-L37	179	81.6
	Alexandra	L10-L19-L18-L17-L16-L15	53	58	L9-L8-L7-L6-L13	145	64	L1-L2-L13	154	87.5
	Thornton	L10-L19-L18-L17-L12-L6	49	50	L9-L8-L7-L6	134	52	L1-L2	143	75.1
	Eildon	L10-L19-L18-L17-L12-L6-L2	60	63	L1	132	62	L9-L8-L3-L6-L3	146	71.6
	Yarra	L10-L21-L24-L25	65	64	L10-L21-L26-L28-L27	67	70	L10-L21-L23-L28-L27	125	75.6
Rubicon	Yea	L43-L4-L13-L42	47	40.6	L43-L4-L13-L39-L40-L42	59	58	L43-L4-L6-L12-L16-L15-L39-L40-L42	72	73
	Alexandra	L43-L4-L13	26	27	L43-L4-L6-L12-L16-L15	39	42	-	-	-
	Thornton	L43-L4	15	15	-	-	-	-	-	-
	Eildon	L43-L4	147	76	L43-L4-L2	26	28	-	-	-
	Yarra	L43-L4-L6-L12-L17-L20-L24-L25	81	88	L43-L5-L8-L11-L19-L21-24-L25	176	107	L43-L4-L13-L41-L42-L36-L29-L27	90	116
